# Western Reserve Takes Important Step in Dental Education

**Published:** 1921-09

**Authors:** 


### WESTERN RESERVE TAKES IMPORTANT STEP IN DENTAL EDUCATION

The Dental School of Western Reserve University announces that, beginning with September 1922, it will require for entrance the completion of one year of work in an acceptable college of arts and sciences in addition to the completion of the regular minimum of fourteen units high school work.

It is believed that the profession will approve this advance in dental education in this school. It will help the profession because it will tend to raise the standards of dentistry. It will help humanity because its health will be safeguarded by men better equipped. Your loyal cooperation means much to us at this time.

In this pre-dental year of college work must be included at least six semester hours of college Chemistry, at least six semester hours of college Biology, or Zoology, at least six semester hours of college English, and an amount of College Physics equivalent to one unit of secondary school Physics, provided the student had not a unit of Physics in the high school. The remainder of the thirty semester hours is to be made up of work in such subjects as the College in which the predental year is taken may prescribe or allow the student to choose.

The tendency to require for entrance to the professional course of the Dental School something more than graduation from the high school has developed in response to the opinion of dental educators that such preliminary requirement will materially improve the curriculum of the dental schools and produce dental graduates of greater power, skill and capability.

Already one state (New York) requires that all men applying for examinations for license in that state, who shall have entered a dental school subsequent to January 1921, must have had a pre-dental year of collegiate training. Fourteen of the forty-three dental schools of the United States will require the pre-dental year of all students entering the first year class in September-October, 1921.

The requirement of the pre-dental year will accomplish several important things that will improve the training given in dental schools.

1. It will relieve the dental curriculum of such subjects as Inorganic Chemistry, Physics, Biology, English, and Technical Drawing. These subjects now occupy somewhat more than one-half of the first year, and their transference to the pre-dental year will allow an extension of the instruction in other subjects of the dental curriculum by from 12 per cent to 16 per cent. This will permit some extension of the fundamental subjects of Anatomy, Histology, Embryology, Physiology, Organic and Physiological Chemistry, Bacteriology and Pathology, and a very material extension, of the various phases of technics, operative and prosthetic dentistry.

2. This change will permit the completion of the fundamental subjects of Anatomy, Histology, Embryology, Organic and Physiological Chemistry, Physiology, Bacteriology, and Pathology, together with the major part of operative and prosthetic technics, by the end of the second year, thus allowing two full years of clinical work, as well as extensions in subjects that are closely correlated with the clinical work like Therapeutics, Radiology, and Dental Pathology. Thus the student will get much more clinical practice than at present and this will be based upon more extensive courses in the fundamental subjects upon which an understanding of clinical practice must be founded.

3. Experience has shown that the character of instruction in the dental school courses is very different from that in high school courses. The result is that the student just out of high school must materially change his methods of study and work and a considerable part of the first year is occupied by the student in finding himself. The character of instruction in the college of arts and sciences is intermediate between that in the high school and that in the dental school. The student who has had the pre-dental year will be far better prepared from the outset to grasp the instruction in professional subjects. That this is true is abundantly proven by the greater facility with which present students who have had a year of college training are able to understand the work of the dental course.

4. The experience in medical education, which over a decade ago went through the same process of advance, shows conclusively that some college work previous to professional studies permits a far better grade of professional teaching and produces graduates of greater capabilities.

